# Management of hyperlipidemia among patients with rheumatoid arthritis in the primary care setting

**DOI:** 10.1186/s12891-015-0700-5

**Published:** 2015-09-03

**Authors:** Kashif Jafri, Lynne Taylor, Melissa Nezamzadeh, Joshua F. Baker, Nehal N. Mehta, Christie Bartels, Catherine T. Williams, Alexis Ogdie

**Affiliations:** Department of Medicine, Perelman School of Medicine at the University of Pennsylvania, Philadelphia, PA USA; Biostatistics Analytic Core, Center for Clinical Epidemiology and Biostatistics, Department of Biostatistics and Epidemiology, Perelman School of Medicine at the University of Pennsylvania, Philadelphia, PA USA; Center for Clinical Epidemiology and Biostatistics, Perelman School of Medicine at the University of Pennsylvania, Philadelphia, PA USA; Division of Rheumatology, Center for Clinical Epidemiology and Biostatistics, Center for Pharmacoepidemiology Research and Training, Perelman School of Medicine at the University of Pennsylvania, White Building, Room 5024, 3400 Spruce St., Philadelphia, PA 19104 USA; Section of Inflammation and Cardiometabolic Diseases, National Heart, Lung, and Blood Institute, Bethesda, MD USA; Division of Rheumatology, School of Medicine and Public Health, University of Wisconsin, Madison, WI USA

## Abstract

**Background:**

Rheumatoid arthritis (RA) has been associated with an increased risk of cardiovascular morbidity and mortality but this has not translated to optimal management of traditional cardiovascular risk factors such as hyperlipidemia. The objectives of this study were to 1) determine the prevalence of screening for hyperlipidemia in patients with RA followed by primary care practitioners (PCP); 2) examine initiation of lipid-lowering therapy in patients with an indication, and 3) assess whether proposed modifications to cardiovascular risk calculations change the percentage of RA patients with an indication for therapy.

**Methods:**

We performed a retrospective cohort study using an academic medical center-based medical record database in the United States. Patients with RA defined by the presence of at least one ICD-9 code between 2005–2010 and followed by a PCP within the health care system were included. The positive predictive value of ICD-9 codes for accurately identifying patients with RA was 96.7 %. Descriptive statistics were used to report the prevalence of screening and use of lipid-lowering therapy among those with an indication. Factors associated with not receiving lipid screening were examined using logistic regression models. Indication for and receipt of therapy were then assessed before and after the application of the European Union League Against Rheumatism (EULAR) recommended multiplier to the Framingham risk score.

**Results:**

Among 1,056 patients with RA followed by PCPs and eligible for lipid screening, lipid screening was ordered for 539 (51 %) within the 3-year follow-up period. Patients with diabetes, hypertension, chronic kidney disease, obesity or age >50 were more likely to be screened. Of those with lipid results (*N* = 290), 25 (9 %) patients had an indication for lipid-lowering therapy based on Adult Treatment Panel III guidelines. Ten (40 %) patients with an indication for lipid-lowering therapy received therapy did not receive therapy. Applying the EULAR multiplier only changed the indication for lipid-lowering therapy in two patients.

**Conclusions:**

Screening and management of traditional cardiovascular risk factors, including hyperlipidemia, need to be optimized.

## Background

Rheumatoid arthritis (RA) is a chronic inflammatory arthritis that is two to three times more common in women and is associated with an increased risk of cardiovascular disease, including myocardial infarction, stroke, and heart failure [1–4]. Although several studies have clearly demonstrated this association, it is unclear whether these observations have impacted the management of cardiovascular risk factors in RA patients in primary care practice [5]. An increased focus on screening for cardiovascular risk factors is especially important given that cardiovascular disease is the leading cause of mortality in RA patients and presents about a decade earlier in these patients compared to the general population [6–8].

The European Union League Against Rheumatism (EULAR) published recommendations in 2010 for the management of traditional cardiovascular risk factors in patients with RA. EULAR advised physicians to calculate a 10-year coronary heart disease (CHD) risk for each patient, to multiply this risk by a factor of 1.5, and to subsequently use the adjusted risk score to manage cardiovascular risk in patients with RA [9]. The treatment of RA as a CHD equivalent has also been proposed to encourage more aggressive cardiovascular risk management [10–12]. Despite these recommendations, one recent study of RA patients receiving Medicare benefits demonstrated that only 45 % were screened for hyperlipidemia [13], and another study of French rheumatology patients revealed that only 20 of 145 patients were receiving statin therapy [14].

Given this preliminary evidence that knowledge of the relationship between rheumatoid arthritis and CHD is not being translated into clinical practice, the present study aimed to assess the management of hyperlipidemia among RA patients in a large retrospective cohort of patients followed by primary care practitioners. More specifically, the study objectives were: 1) determine the prevalence of screening for hyperlipidemia among patients with RA and describe the characteristics of patients least likely to be screened, 2) examine whether Adult Treatment Panel (ATP) III guidelines for the initiation of lipid-lowering therapy are being followed among those patients who receive lipid screening, and 3) assess whether proposed modifications to risk calculations change the percentage of RA patients that should be receiving lipid-lowering therapy.

## Methods

### Study design and setting

We performed a retrospective cohort study among patients with RA followed by a primary care physician in the University of Pennsylvania Health System (UPHS) between January 2005 and February 2010. Only data from this time period was included in the study. This time period was chosen for the following reasons: a) most primary care practices at Penn began using the electronic medical record in 2004, b) evidence of increased cardiovascular risk among patients with RA was reported in the literature during this period, and c) EULAR guidelines for the management of cardiovascular risk in RA were published in February 2010.

### Data source

The Penn Data Store (PDS) integrates electronic medical record data from multiple systems within the University of Pennsylvania Health System. It includes all discrete data including diagnostic codes, medications, laboratory and imaging orders, and most laboratory results within the inpatient and outpatient medical record systems.

### Study population

Rheumatoid arthritis patients were defined by the presence of at least one International Classification of Diseases ninth edition (ICD-9) code consistent with RA (ICD9 714.0-714.33) in the electronic medical record. Cohort entry occurred at the first RA code. Inclusion criteria were age >18 at cohort entry and the receipt of medical care from a primary care physician (internal medicine, geriatrics, or family medicine) or physician extender (nurse practitioner or physician assistant) within UPHS, including two or more outpatient visits and one year of follow-up time. Follow-up time ended after a maximum of 3 years from cohort entry. Patients were excluded if they were less than 18 years of age at cohort entry or if they were not followed by a primary care practitioner within UPHS. We additionally excluded patients on a lipid lowering agent at the time of cohort entry.

### Validation of the exposure

We first performed a validation study to ensure accurate identification of patients with RA using a single ICD-9 code. Sixty charts were manually reviewed to confirm the diagnosis of RA using a rheumatologist’s diagnosis as the gold standard. The positive predictive value of a single ICD-9 code for RA was 96.7 % (95 % CI: 0.885-0.996).

### Outcomes

We examined several outcomes. The first primary outcome was receipt of an order for lipid screening in the three years following the first code for rheumatoid arthritis. Lipid screening was defined as ordering of high-density lipoprotein (HDL) and total cholesterol, or a lipid panel by the physician. Next, we examined the prevalence of an indication for therapy determined by ATP III guidelines that required calculation of the Framingham Score and presence of a low-density lipoprotein (LDL) result (http://www.nhlbi.nih.gov/health-pro/guidelines/in-develop/cholesterol-in-adults) [15]. LDL cutoffs, derived from the ATP III guidelines, were ≥190 mg/dl for risk category 0, ≥160 mg/dl for risk category 1, and ≥130 mg/dl for risk categories 2 and 3. The determination of whether there was an indication for therapy occurred only in those with complete data. We then examined receipt of lipid-lowering medication prescriptions in those that had an indication for such therapy in the one year following the first set of lipid results. As we used medical record data, the presence of the prescriptions was evidence of physician intent to prescribe the medication. The medical record does not contain information on whether the prescription was filled. Prescribing of a lipid-lowering therapy was defined as the presence of a prescription of any of the following: fenofibrate, gemfibrozil, colesevelam, cholestyramine, ezetimibe, niacin, fish oil, omega 3 fatty acids, atorvastatin, fluvastatin, lovastatin, pravastatin, rosuvastatin, and simvastatin. Finally, we applied the EULAR multiplier (1.5 × the Framingham risk score), recalculated the ATP III category and the indication for therapy, and then examined the prevalence of therapy receipt within each category. Of note, the EULAR recommendation suggests use of this multiplier in patients meeting two of the following criteria: a) disease duration of more than ten years, b) rheumatoid factor or anti-cyclic citrullinated peptide positivity, and c) presence of extra-articular manifestations [9]. Given the difficulty of extracting this data without individual chart reviews, we applied the multiplier to all patients in the cohort.

### Covariates

The following covariates were assessed in the one year prior to and including cohort entry: age, gender, race, socioeconomic status based on average income in the patient’s census track, smoking, alcohol intake, obesity, hypertension, previous diagnosis of hyperlipidemia, diabetes, previous cardiovascular disease (specifically myocardial infarction, stent placement, transient ischemic attack, and cerebrovascular disease), comorbidities including chronic kidney disease and peripheral arterial disease, and medications used for RA including biologic and non-biologic disease-modifying anti-rheumatic drugs (DMARDs), non-steroidal anti-inflammatory drugs (NSAIDs), and corticosteroids. Additionally, we examined contraindications for lipid-lowering therapy in the year prior to the first lipid results. These included myopathy, pregnancy, liver disease, and use of medications with interactions including erythromycin, protease inhibitors, itraconazole, and clarithromycin. All medical diagnoses were identified by the presence of ICD-9 codes for that diagnosis. The Framingham risk score was calculated as previously described and required the presence of the following variables: age, gender, high-density lipoprotein (HDL), total cholesterol, systolic blood pressure, use of anti-hypertensive medications, and tobacco use [15]. Risk categories were generated based on Framingham risk scores using ATP III guidelines (http://www.nhlbi.nih.gov/health-pro/guidelines/current/cholesterol-guidelines/final-report). Adjusted risk categories were similarly based on adjusted Framingham risk scores (multiplied by 1.5) using ATP III guidelines.

### Statistical analysis

Percentages were used to describe the prevalence of RA patients that received lipid screening, the prevalence of RA patients with an indication for lipid-lowering therapy, and the prevalence of lipid-lowering therapy receipt within the different risk categories. Percentages were also employed to characterize the number of patients with an indication for lipid-lowering therapy after application of the EULAR recommended multiplier. We used logistic regression models to examine the association of baseline covariates with receipt of screening. We first examined univariable logistic regression models and then performed multivariable logistic regression modeling using only the covariates that were significant at the univariable modeling stage. Covariates were retained in the final model if they were significantly associated with receipt of screening (*p* < 0.1) after adjusting for the other covariates. The final model’s c statistic was used to describe the model’s ability to predict not being screened, and the covariates’ OR and 95%CI were used to describe the likelihood of not being screened.

### Ethics approval

This study was approved by the University of Pennsylvania Institutional Review Board. As only existing de-identified data was analyzed, a waiver of written informed consent was obtained. Children were not included in the study. This manuscript was prepared in accordance with the Strobe statement [16].

## Results

The study population consisted of 1418 patients with rheumatoid arthritis. Table 1 provides the baseline characteristics of this population. The patients were predominantly female (85 %) and of age greater than 50 years (65 %). Among those eligible for screening (*N* = 1056 patients not on a lipid lowering therapy at entrance into the cohort), lipid testing was ordered in 539 (51 %) patients within the 3-year follow-up period, although not all patients complied with the request for screening. There was not a significant association between gender and the performance of lipid screening. Lipid screening was more likely to be performed in patients of age 50 years or more (adjusted odds ratio; aOR 1.68, 95 % CI: 1.29-2.18). Additionally, patients with hypertension (aOR = 2.12, 95 % CI: 1.35-3.32), diabetes (aOR 2.06, 95 % CI: 1.48-2.87), obesity (aOR 2.52, 95 % CI:1.51-4.19) were more likely to be screened (Table 2).

**Table 1:** Patient Demographics (*n* = 1056)

		N (%)
Sex (Female)		893 (85 %)
Age	Mean (SD)	55.1 (15.9)
	Age <50 years	369 (35 %)
Race	White	401 (38 %)
	Black	550 (52 %)
	Asian	23 (2 %)
	Other	58 (5 %)
	Unknown/Missing	24 (2 %)
Diagnoses and Risk Factors		
	Alcohol use	141 (13 %)
	Missing Alcohol Status	709 (67 %)
	Tobacco use	71 (7 %)
	Missing Tobacco Status	708 (67 %)
	Hypertension^a^	109 (10 %)
	Hyperlipidemia^a^	16 (2 %)
	Diabetes mellitus^a^	201 (19 %)
	Chronic kidney disease^a^	13 (1 %)
	Cardiovascular disease^a^	7 (1 %)
	Obesity^a^	85 (8 %)
Body Mass Index Categories	Underweight (<18.5)	11 (<1 %)
	Normal (18.5-24.9)	87 (6 %)
	Overweight (25–29.9)	102 (7 %)
	Obese (≥30)	130 (9 %)
	Missing	1,088 (77 %)
Medication Use		
	NSAIDs	271 (26 %)
	DMARDs	330 (31 %)
	Corticosteroids	199 (19 %)

All variables assessed at cohort entrance

^a^Defined by ICD9 code

*Abbreviations*: *NSAIDs* Non-steroidal anti-inflammatory drugs, *DMARDs* Disease Modifying Antirheumatic Drugs

**Table 2:** Logistic regression for receipt of screening

		Univariable	Final Multivariable Model^a^
		OR (95 % CI)	OR (95 % CI)
Age	≥50 versus <50	1.69 (1.31-2.18)	1.68 (1.29-2.18)
Sex	Female vs Male	0.94 (0.68-1.33)	
Race	Caucasian	Ref	
	Black or African American	1.31 (1.01-1.69)	
	Asian	0.69 (0.29-1.62)	
	Other or unknown	0.74 (0.45-1.21)	
Hypertension		2.65 (1.72-4.10)	2.12 (1.35-3.32)
Hyperlipidemia Diagnosis		1.61 (0.58-4.46)	
Diabetes mellitus		2.22 (1.61-3.07)	2.06 (1.48-2.87)
Obesity		2.62 (1.61-4.27)	2.52 (1.51-4.19)
BMI^a^		1.04 (1.01-1.07)	
BMI Category^a^	Normal (18.5-24.9)	REF	
	Overweight (25–29.9)	1.66 (0.93-2.96)	
	Obese (≥30)	2.17 (1.24-3.78)	
	Underweight (<18.5)	0.40 (0.10-1.62)	
Peripheral Arterial Disease		1.92 (0.17-21.3)	
Tobacco use	Current smoker vs non-smoker or past-smoker (*n* = 348)	0.70 (0.41-1.18)	

The c-statistic (equivalent to area under the curve) for the model was 0.63 for the association between the predicted probabilities and observed responses for the final multivariable model

^a^Given the large amount of missing data for BMI and the risk for selection bias in using a complete case analysis, we have instead used a binary variable for obesity identified using ICD9 codes. The OR of 1.04 is for each unit increase in BMI

**Fig. 1:**
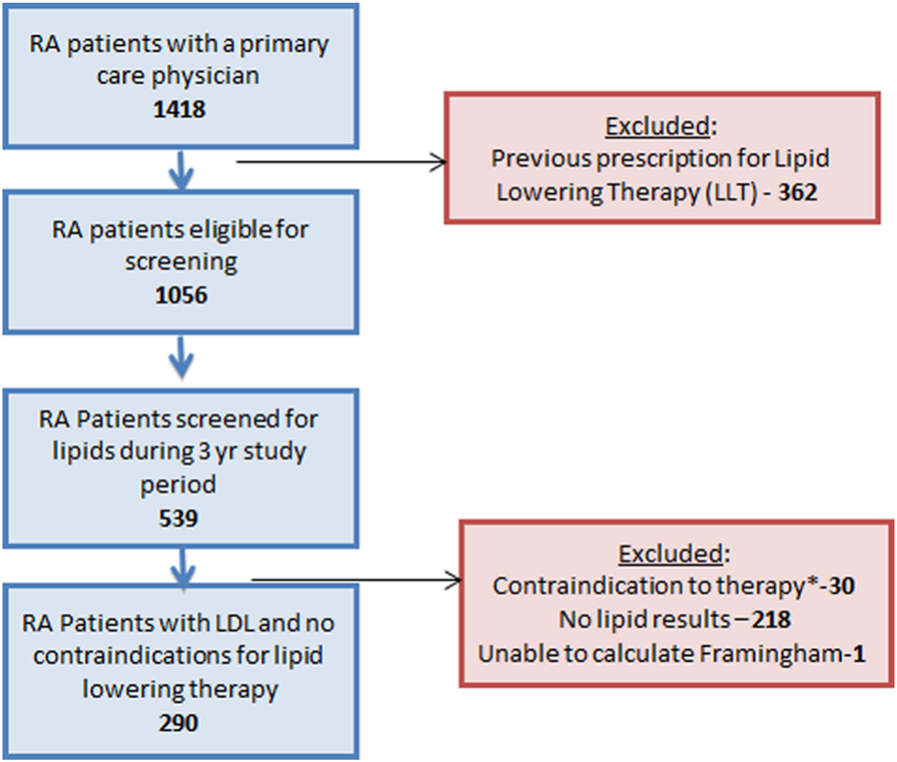
Flow Diagram. Among 1418 patients with rheumatoid arthritis followed by a primary care physician, 1056 were eligible for screening and 539 received screening. Among those with orders for lipids, 290 had complete lipid panels for analysis after excluding those with contraindications to therapy. *Contraindications to therapy included pregnancy (*N* = 5), myopathy (*N* = 2), liver disease (e.g. cirrhosis, liver cancer, alcoholic liver disease, hepatitis C, hepatitis B) (*N* = 16) or interacting medications including erythromycin, protease inhibitors, itraconazole, and clarithromycin (*N* = 8). Abbreviations: RA = rheumatoid arthritis, LDL = low density lipoprotein, LLT = lipid lowering therapy

Among patients in whom lipid testing was recommended (*N* = 593), 290 patients had available lipid results after excluding patients with a contraindication to lipid-lowering therapy (*N* = 30), and those without sufficient data to calculate a Framingham risk score (*N* = 1) (Fig. 1). Most patients (65 %) were in the lowest risk category (0–1 cardiovascular risk factors), and 32 % of patients were in the highest risk group with a 10-year cardiovascular risk greater than 20 %. Very few patients (3 %) had 2 or more cardiovascular risk factors with a 10-year cardiovascular risk less than 20 %. Among the 290 patients with complete data, the mean total cholesterol was 190.2 mg/dl (SD 42.2), the mean LDL was 109.4 mg/dl (SD 32.4), and the mean HDL was 58.2 mg/dl (SD 20.3). Mean age at the time of the lipid panel was 61.4 years (SD 13.5).

When the ATP III guidelines were applied to this group to determine whether a lipid-lowering medication was indicated based on the patient's LDL value (Table 3), 25 (9 %) of patients had an indication for therapy, and of those with an indication, 14 (56 %) received lipid-lowering therapy. Among all patients prescribed lipid-lowering therapy (with or without an indication based on ATP III), 53 were prescribed a statin, 3 were prescribed a fibrate, and 15 were prescribed another class of lipid-lowering medication. After the EULAR multiplier was applied to the Framingham risk scores, 3 patients shifted from the lowest risk category to the highest risk category, and the number of patients with an indication for therapy increased from 25 to 27. The percentage of patients with an indication for therapy that received lipid-lowering therapy remained the same (56 %).

**Table 3:** ATP III Risk Category, Indication for Lipid Lowering Therapy, and Receipt of Therapy (*n* = 290 patients with lipid results)

	Before EULAR Adjustment		After EULAR Adjustment	
Risk Category	N (%)	Indication for therapy based on LDL	N (%)	Indication for therapy after adjustment
		N (%)		N (%)
CHD or CHD Risk Equivalents	92 (32 %)	20 (22 %)	96 (33 %)	23 (24 %)
OR				
10 yr risk >20 %				
2+ Risk Factors AND	7 (1 %)	1 (50 %)	1 (0.3 %)	0 (0 %)
10-yr risk 10-20 %				
2+ Risk Factors AND	2 (2 %)	0 (0 %)	7 (2 %)	0 (0 %)
10-yr risk <10 %				
0-1 Risk Factors AND	189 (65 %)	4 (2 %)	186 (64 %)	4 (2 %)
10-yr risk <10 %				
Total	290 (100 %)	25 (9 %)	290 (100 %)	27 (9 %)

Indication for therapy in each risk category was based on the ATP III guidelines which suggest the following LDL cutoffs: 190 mg/dl for risk category 0, ≥160 mg/dl for risk category 1, and ≥130 mg/dl for risk categories 2 and 3.(15)

## Discussion

Given the high burden of cardiovascular disease in patients with RA, there has been increased emphasis on understanding physician screening practices for cardiovascular risk factors in this patient population [9]. The present study demonstrated that 51 % of patients received an order for lipid screening within the 3-year follow-up period. Screening was significantly less likely to be performed in patients with an age less than 50; however, those with concomitant hypertension or diabetes were more likely to be screened, suggesting attention to traditional risk factors. While the majority of patients (65 %) in this study had 0–1 cardiovascular risk factors, 32 % of patients were in the high-risk category with a 10-year cardiovascular risk greater than 20 %. Approximately half of the patients with an indication for lipid-lowering therapy based on their lipid screening results did not receive an appropriate pharmacologic treatment during the study period. Interestingly, the percentage of patients that did not receive indicated lipid-lowering therapy remained essentially the same after applying the EULAR-recommended risk adjustment.

This study contributes to the growing body of literature that reflects inadequate cardiovascular screening practices and suboptimal rates of indicated therapeutic interventions to modify cardiovascular risk in RA [13, 17]. Our results regarding inadequate screening mirror those of the Medicare population [13] and those in Rochester, MN [17]. In our study, we were additionally able to observe screening practices in younger patients with RA and to consider important contraindications to lipid-lowering therapy. Other strengths of this study include the use of a medical record database with access to physician notes that aided in forming a well-defined population and access to lipid values that allowed for understanding the effect of the EULAR multiplier on indications for therapy.

The findings in this study have important implications for the management of patients with RA. Only 51 % of the patients were screened for lipid levels within the 3-year follow-up period, and only 56 % received appropriate lipid-lowering therapy within 1 year of being screened. Possible quality improvement measures include the education of primary care physicians about the importance of preventive cardiovascular care in patients with RA and more effective coordination of care among rheumatologists, cardiologists, and primary care physicians in light of the complexity of this patient population. Enhancing awareness of cardiovascular risk in RA among all care providers would ensure that collective responsibility is taken for appropriate primary prevention measures and potentially increase the frequency of lipid screening. By increasing awareness of the importance of lipid screening in RA, we can translate our knowledge about the relationship between RA and cardiovascular disease into more aggressive cardiovascular risk management in clinical practice.

In this study, we found that the EULAR modification of the cardiovascular risk score did not significantly change the number of patients who had an indication for therapy. The EULAR recommendations suggested multiplying the SCORE or Framingham risk score by a factor of 1.5 in patients with a) disease duration of more than ten years, b) rheumatoid factor (RF) or anti-cyclic citrullinated peptide antibiody (ACPA) positivity, and c) presence of extra-articular manifestations [9]. We did not have sufficient information about disease duration, antibody status or extra-articular manifestations so we applied the 1.5-multiplier to all patients. This should have resulted in more changes than anticipated, thus, the lack of significant change was interesting. A similar report by Gomez-Vaquero et al. showed little change in the SCORE algorithms, although a more recent paper by Rosales-Alexander reported the EULAR multiple did in fact meaningfully change the risk assessment for patients with an intermediate risk using SCORE alone [18, 19]. The EULAR multiplier was derived from expert opinion based on the mean standardized mortality rate of 1.9 for cardiovascular mortality among patients with established disease [9]. The 1.5 multiplier was felt to be conservative compared to the 1.9. At the time these recommendations were developed, little evidence existed about measurement of cardiovascular risk among patients with RA. Since that time, a handful of studies have now indicated that the Framingham and SCORE risk scores underestimate true cardiovascular risk [5, 20–22]. One reason for this is that inflammation, likely a major contributor to the development of atherosclerosis in patients with RA, is not included [23]. Additional studies are needed to develop better models for cardiovascular risk stratification specific to patients with RA [24].

Limitations of the present study should also be recognized. The study was performed at a single center, although the data is likely generalizable to other large urban academic institutions that serve low-income populations. The medical record database used in this study contained incomplete data on cardiovascular risk factors, including smoking and body mass index, for some patients. For instance, if smoking status or alcohol status were not recorded, they were assumed to be negative; the absence of such data would likely represent an underestimation of cardiovascular risk. Furthermore, the exclusion of patients without complete lipid panels may have resulted in a selection bias and may reflect the patterns of certain physicians to record complete data; it may also reflect insurance coverage since the laboratory tests for some patients may not have been performed within the UPHS system. Additionally, we did not perform detailed chart reviews for each patient so information on lifestyle modification and recommendations for hyperlipidemia were not captured. Finally, screening practices of primary care physicians were specifically investigated because they are generally responsible for providing basic preventive cardiovascular care. Nevertheless, patients could potentially have received lipid screening and prescriptions for lipid-lowering therapy from outside providers without accurate documentation in our medical record database. Similarly, only approximately one third of patients were recorded as having a DMARD recorded during the baseline period. While this is consistent with other population-based estimates of DMARD use among patients with RA in the United States, prescriptions from outside rheumatologists could have been missed [25, 26]. However, during this time period, there was a system-wide emphasis placed on medication reconciliation requiring this to be performed at each visit.

Systematic studies of the barriers to lipid screening and to patient adherence with lipid-lowering therapy will be essential in optimizing long-term outcomes for RA patients. Although the increased cardiovascular risk in RA may be mediated by both traditional risk factors and additional risk factors such as systemic inflammation and a prothrombotic state [27] a fundamental principle of RA management should be the modification of traditional risk factors with the appropriate prescription of lipid-lowering therapy. Both before and after the EULAR adjustment of the ATP III risk categories, about one-half of the patients in this study did not receive a basic therapeutic intervention that was indicated to reduce their cardiovascular risk. A recent large population-based study of RA patients with incident statin use demonstrated that statin discontinuation was associated with a 60 % increased risk of cardiovascular mortality and a 79 % increased risk of all-cause mortality [28]. Although it is known that statins have both cholesterol-lowering effects and potent anti-inflammatory properties, the “healthy adherer effect” (i.e., more compliant patients engage in healthier behaviors) has also been postulated to explain the association of statin discontinuation with poorer outcomes such as increased risk of acute myocardial infarction [29] (17). The mechanisms by which statins influence morbidity and mortality in RA need to be characterized more specifically, and appropriately powered prospective studies should be designed to address the potential for statins to reduce cardiovascular and all-cause mortality in RA. An analysis of the effects of statins on cardiovascular risk in this patient population will be particularly important given that inflammatory arthritis is not included in the cardiovascular risk calculator recently published by the American College of Cardiology (ACC) and American Heart Association (AHA) [30].

## Conclusions

In summary, these data highlight the lack of screening for hyperlipidemia in approximately half of patients with rheumatoid arthritis and the under-treatment of a similar percentage of patients who had an indication for lipid-lowering therapy based on ATP III guidelines prior to publication of the EULAR recommendations for cardiovascular risk management. Regardless of whether primary care physicians continue to use the ATP III guidelines or widely adopt the 2013 ACC/AHA guidelines regarding the use of lipid-lowering therapy, lipid screening is a prerequisite for the appropriate assessment of cardiovascular risk. Further research is needed to address the effectiveness of interventions to improve lipid screening in patients with rheumatoid arthritis and also to develop a systematic approach to characterizing cardiovascular risk in this patient population.

## Abbreviations

ACC: American College of Cardiology
AHA: American Heart Association
ATP III: Third Report of the Expert Panel on Detection Evaluation, and Treatment of High Blood Cholesterol in Adults (Adult Treatment Panel III)
CHD: coronary heart disease
DMARDs: disease-modifying anti-rheumatic drugs
EULAR: European Union League Against Rheumatism
HDL: high-density lipoprotein
ICD-9: International Classification of Diseases ninth edition
LDL: low-density lipoprotein
NSAIDs: non-steroidal anti-inflammatory drugs
PDS: Penn Data Store
RA: Rheumatoid Arthritis
UPHS: University of Pennsylvania Health System
